# Calreticulin-STAT3 Signaling Pathway Modulates Mitochondrial Function in a Rat Model of Furazolidone-Induced Dilated Cardiomyopathy

**DOI:** 10.1371/journal.pone.0066779

**Published:** 2013-06-20

**Authors:** Ming Zhang, Jin Wei, Hu Shan, Hao Wang, Yanhe Zhu, Jiahong Xue, Lin Lin, Rui Yan

**Affiliations:** 1 Department of Cardiology, The Second Affiliated Hospital, Xi’an Jiaotong University School of Medicine, Xi’an, Shaanxi, China; 2 Department of Anesthesiology, Wake Forest University School of Medicine, Winston-Salem, North Carolina, United States of America; University of Otago, New Zealand

## Abstract

**Background:**

Calreticulin is a Ca^2+^-binding chaperone of the endoplasmic reticulum which regulates the signal transducer and activator of transcription 3 (STAT3). The effects of the calreticulin-STAT3 signaling pathway on cardiac mitochondria and on the progress of dilated cardiomyopathy (DCM) are still unclear.

**Methods and Results:**

The DCM model was generated in rats by the daily oral administration of furazolidone. Echocardiographic and hemodynamic studies demonstrated enlarged LV dimensions and reduced systolic and diastolic functions at thirty weeks after the first furazolidone administration. Morphometric analysis showed significant myocardial degeneration, interstitial fibrosis, and mitochondrial swelling with fractured or dissolved cristae in the model group. Compared with the control group, the mitochondrial membrane potential (MMP) level of the freshly isolated cardiac mitochondria and the enzyme activities of cytochrome c oxidase and succinate dehydrogenase in the model group were significantly decreased (*P*<0.05). Real-time PCR and western-blot revealed the increased expression of calreticulin associated with decreased activity of STAT3 in the model group. When cultured neonatal rat cardiomyocytes were exposed to furazolidone, a dose-dependent decrease in cell viability and MMP, and the increase of apoptosis rate were observed. The mRNA and protein expression of CRT gradually increased with the increase of furazolidone concentration, associated with a gradual decrease of the STAT3 phosphorylation level both in the whole cell and mitochondrial fraction. When calreticulin was knocked down with siRNA in cardiomyocytes, these changes of cardiomyocytes and mitochondria induced by furazolidone were significantly attenuated.

**Conclusions:**

A rat model of DCM induced by furazolidone is successfully established. The calreticulin-STAT3 signaling pathway is involved in cardiac mitochondrial injury and the progress of furazolidone induced DCM.

## Introduction

Dilated cardiomyopathy (DCM) is the most common cardiomyopathy worldwide characterized by left ventricle (LV) dilation associated with systolic dysfunction, diastolic dysfunction and impaired right ventricular function. However, the mechanisms and pathogenesis of DCM are still poorly understood [1].

Calreticulin (CRT) is a Ca^2+^-binding chaperone of the endoplasmic reticulum involved in Ca^2+^ storage and modulation of intracellular Ca^2+^ homeostasis [2]. CRT is highly expressed in the embryonic heart, but immediately down-regulated after birth [3]. The overexpression of CRT in the postnatal heart results in cardiomyopathy-like phenotypes including decreased systolic function, chamber dilation, arrhythmia, and sudden death [4], [5]. However, the alterations of CRT expression and its roles in DCM remain ambiguous [6], [7].

The signal transducer and activator of transcription 3 (STAT3), one downstream signaling molecule of CRT, plays an important role in the progress of cardiomyopathy. Many studies have shown that the activation of STAT3 promotes cardiomyocyte survival and hypertrophy, as well as cardiac angiogenesis, in response to various pathophysiologic stimuli, strongly suggesting that STAT3 is beneficial for the heart [8]. Moreover, one previous study shows that the protein expression of STAT3 is down-regulated in the patients with end-stage of DCM [9], while overexpression of STAT3 in the heart protects against doxorubicin-induced cardiomyopathy [10]. Further study suggests that the activation of STAT3 protects cardiomyocytes from hypoxia/reoxygenation-induced damage, mainly through its induction of the expression of manganese superoxide dismutase (MnSOD) [11], which is important to mitochondria due to its ability on scavenging reactive oxygen species.

CRT and STAT3 molecules play important roles in the cardiovascular diseases, but the role of the CRT-STAT3 signaling pathway in the progress of DCM is little understood. Although previous studies have demonstrated that CRT is an upstream regulator of STAT3 [12], the regulatory effect of CRT on STAT3 is still unclear. Du and colleagues have demonstrated that CRT as an upstream regulator of STAT3 promotes cell motility and enhances resistance to anoikis in esophageal squamous cell carcinoma [13], while another study has shown that CRT is an upstream signaling molecule of STAT3, and inhibits STAT3 signaling and function [12].

In order to determine the processes and mechanisms involved in DCM pathogenesis, many kinds of animal models have been established [14]. Furazolidone (FZD), a synthetic nitrofuran with a board spectrum of antimicrobial activities, is commonly used to establish a turkey poult model of DCM [15]. Due to the similarities of DCM in turkey poults to DCM in human [15], the cellular and molecular mechanisms of DCM have often been investigated in the turkey poult model of DCM induced by FZD [16], [17]. However, the rats are more widely used in the field of medical research, and allow for a range of experimental approaches including echocardiography to confirm the presence of DCM in vivo, a rat model of DCM induced by FZD was established in our present study. CRT-STAT3 signaling pathway and its effects on the cardiac mitochondria have also been studied in this FZD induced rat DCM model.

## Materials and Methods

### Reagents

FZD (C_8_H_7_N_3_O_5_, MW 225.16, 99%), carboxy methylcellulose-Na, dimethyl sulphoxide (DMSO), and 3-(4,5-dimethylthiazolyl-2)-2,5-diphenyltetrazolium bromide (MTT) were all purchased from Sigma. 5-bromo-2-deoxyuridine (5-BrdU), trypsin, and collagenase type II were purchased from Amresco.

### Animal Model

Thirty-five male Sprague-Dawley rats (40–60 g) provided by the Animal Center of Xi’an Jiaotong University, were randomly divided into three groups based on the treatments as follows: (1) untreated group: rats drank tap water only; (2) control group: rats drank 0.15% carboxy methylcellulose-Na solution dissolved in tap water; (3) model group: rats were administrated FZD solution dissolved in 0.15% carboxy methylcellulose-Na solution (700 ppm) [15]. All animal study protocols were approved by the Institutional Animal Research and Ethics Committee of Xi’an Jiaotong University (SCXK2007-001).

### Echocardiographic Studies

Echocardiographic studies were performed by an investigator blinded to the treatments. Two-dimensional, targeted M-mode tracings were obtained at the level of the papillary muscles with an echocardiographic system (Philips iE33) equipped with a 12-4 MHz transducer (Philips). A series of cardiac parameters including a Tei index [18], were measured and calculated. Each measurement was obtained with an average of three consecutive heart beats.

### Electrocardiogram and Hemodynamic Studies

After anesthetizing rats by intraperitoneal injection of chloral hydrate, electrocardiograms of the rats were recorded using the BL-420E Biologic Function Determining System (TME Technology Co. Ltd, China), and the electrocardiogram intervals and segments were accurately measured. The QαT interval defined as the interval from the R wave to the apex of the T wave [19], was used as an approximation of electrical systole, rather than the Q-T interval, because of the difficulty in identifying the termination of the T wave in the rat electrocardiogram. Then a PE-50 catheter connecting a pressure-electricity transducer was inserted into the right carotid artery and advanced into the left ventricular (LV) to record LV systolic and diastolic pressures, as well as the maximum and minimum rates of LV pressure development (dP/dt), using the Power Lab 4.12 system (AD instrument, Sydney, Australia).

### Histology and Morphometric Analysis

The LV myocardia were fixed in neutral 10% buffered formalin, and paraffin sections were cut. The paraffin sections were stained with hematoxylin-eosin and observed using optical microscopy. In order to detect fibrosis in cardiac muscle, the paraffin sections were stained with Masson’s trichrome. After each field was scanned and computerized with a digital image analyzer Lecia Qwin550CW (Lecia Company, Germany), the collagen volume fraction was calculated as the sum of the total area of collagen in the entire visual field divided by the sum of total connective tissue area plus the myocardial area in the entire visual field.

Fresh tissue from the apex of the hearts was cut into pieces 1 mm^3^, fixed with 2.5% glutaraldehyde, then fixed with 1% perosmic acid and dehydrated in an ethanol series. Ultrathin sections were placed on 400 mesh grids and double-stained with uranyl acetate and lead citrate, then observed with a transmission electron microscope (HITACHI-H7650, Tokyo, Japan). Five ultrathin sections from each rat were selected at random for further stereological analysis by the transmission electron microscopy. Ten microscopic fields were randomly chosen per section and photographed. Using a computer imaging analysis system, the reference system (cytoplasm and mitochondrion) area, number of mitochondria cross-section, mitochondrial perimeter, and mitochondrial long axis and short axis were measured. Based on the stereological principles for morphological study [20], mitochondrial number per unit area (N), average diameter (D), volume density (Vv), surface density (Sv), numerical density (Nv) and specific surface (Rsv, surface-to-volume ratio) of mitochondria were all further calculated.

### Cell Culture

Neonatal rat cardiomyocytes (NRCMs) were prepared as described elsewhere with some modifications [21]. Briefly, hearts harvested from 1 to 2 days old Sprague-Dawley rat were washed in PBS (Mg^2+^, Ca^2+^ free). Then hearts were minced and digested with 0.1% trypsin and 0.05% collagenase type II at 37°C. The supernatant was collected in DMEM/F12 medium (Hyclone) containing 10% heat-inactivated fetal bovine serum (FBS, Gibco) and the digestion steps were repeated up to 12 times. Cardiomyocytes were purified from fibroblasts by differential adhesiveness. These cardiomyocytes were cultured finally in DMEM/F12 medium, containing 10% FBS, 100 units/ml penicillin G and 100 µg/ml streptomycin sulfate at 37°C in a humidified atmosphere containing 5% CO_2_ and 95% air. The compound 5-BrdU (0.1 mmol/L) was added during the first 48 h to inhibit the proliferation of non-cardiomyocytes. More than 90% of cells were cardiomyocytes (positive for α-actinin and beating features). Before treatment, the cells were serum starved for 24 h in DMEM/F12 medium containing 0.5% FBS. For each experiment in vitro, FZD was first dissolved in DMSO and then with specific culture medium to the final concentration of which the overall DMSO concentration was 0.1% (v/v). Therefore 0.1%DMSO was used as a control for cell culture.

### siRNA Transient Transfection

CRT siRNA was introduced into the NRCMs using DharmaFECT1 (Dharmacon, T2001-01) according to the manufacturer’s instructions. Forty-eight hours after transfection, the cells were harvested. Lysates were prepared for analysis by RT-PCR and western blot to evaluate the silencing effect of CRT siRNA. The CRT siRNA were purchased from GenePharma Co. Ltd (Shanghai, China). The target sequence for CRT was 5′-CUGGGUCGAAUCCAAACAUTT-3′ (sense) and 5′-AUGUUUGGAUUCGACCCAGTT-3′ (antisense). A nonspecific control siRNA was used as a negative control.

### MTT Assay

Cells were inoculated into a 96-well plate and cultured for 48 h under the FZD incubation. Then the medium was removed and MTT solution (0.5 mg/ml) was added to each well. The cells were incubated in a 5% CO_2_ incubator at 37°C for an additional 4 h. MTT solution was replaced by DMSO to dissolve blue formazan crystals, and absorbance was measured at 490 nm using a microplate reader.

### Apoptosis Assay

Cell apoptosis was detected with flow cytometry using the Annexin V-FITC apoptosis detection kit (Merck KGaA, Darmstadt, Germany). Briefly, cells were washed with PBS, detached with 0.25% trypsin, resuspended in 500 µl of binding buffer (1×10^6^ cells/ml) and incubated with the FITC-conjugated annexin V antibody and propidium iodide for 20 min at room temperature. Annexin V binding was analyzed by FACScan (Becton Dickinson GmbH, Heidelberg, Germany), collecting the fluorescence of 10000 cells according to the manufacturer’s instructions. Cell apoptosis was expressed as percentages of annexin V-FITC-positive cells compared to the total.

### Isolation of Cardiac Mitochondria

Mitochondria were isolated from the whole rat ventricles and neonatal rat cardiomyocytes by differential centrifugation. Briefly, nuclei and unbroken cells were pulled down by centrifugation at 1000 g for 10 min at 4°C. Then, the mitochondrial fraction was obtained by centrifugation of supernatant at 10000 g for 10 min at 4°C, suspended in mitochondrial storage fluid (300 mmol/L sucrose, 2 mmol/L Hepes, 0.1 mmol/L EGTA, pH7.4), and stored at −80°C.

### JC-1 Assay for Mitochondrial Membrane Potential

Mitochondrial membrane potential (MMP) was assessed using the lipophilic cationic probe 5,5′,6,6′-tetrachloro-1,1′,3,3′-tetraethylbenzimidazolylcarbocyanine iodide (JC-1). For quantitative fluorescence measurements, isolated fresh mitochondria and cells were rinsed once after JC-1 staining and scanned with a fluorescence microplate reader (Tecan Infinite M200, Switzerland) at 488 nm excitation and 535 and 590 nm emission to measure green and red JC-1 fluorescence, respectively. Each well was scanned by measuring the intensity of each of 25 squares (of 1 mm^2^ area) arranged in a 5×5 rectangular array.

### Enzyme Activities Measurement

Cytochrome c oxidase (COX) and succinate dehydrogenase (SDH) activities were measured using a COX assay kit (Genemed Scientifics Inc., Shanghai, China) and SDH assay kit (Jiancheng Bioengineering Co., Nanjing, China) following the manufacturer’s instructions. Briefly, COX activity was quantitatively determined due to the changes of absorbance at 550 nm under the spectrophotometer, where cardiac mitochondria protein catalyzed reduced cytochrome c into oxidized cytochrome c. When cardiac mitochondria protein catalyzed the substrates, FAD was reduced to FADH, coupled with a 2,6-DPIP reduction reaction, so that SDH activity was further calculated according to the speed of 2,6-DPIP reduction reaction.

### Real-time PCR

The total RNA was extracted using the TRIzol reagent (Invitrogen) and reverse transcription was carried out using an RT-PCR kit (TaKaRa). Real-time PCR was performed with SYBRExScript™ RT-PCR Kit (TaKaRa) on an iQ5 Multicolor Real-Time PCR Detection System (Bio-Rad, Hercules, CA) according to the manufacturer’s protocol. The primers were as follows: atrial natriuretic peptide (ANP), 5′-TACAGTGCGGTGTCCAACA-3′ (forward) and 5′-GTTGACTTCCCCAGTCCAG-3′ (reverse); brain natriuretic peptide (BNP), 5′-ATTCTGCTCCTGCTTTTCC-3′ (forward) and 5′-GCCTTGGTCCTTTGAGAG-3′ (reverse); CRT, 5′-TTCTTGGACGGAGATGC-3′ (forward) and 5′-CATCTTGGCTTGTCTGC-3′ (reverse); STAT3, 5′-GTAGTGACGGAG AAGCAG-3′ (forward) and 5′-TCACAGACTGGTTGTTTCC-3′ (reverse); MnSOD, 5′-GCATTTTCTGGACAAACC-3′ (forward) and 5′-GACTCCCACAGACACAGC-3′ (reverse); and glyceraldehyde-3-phosphate dehydrogenase (GAPDH), 5′-TTGTGATGGGTGTGAACC-3′ (forward) and 5′-TTCTGAGTGGCAGTGATG-3′ (reverse). The comparative CT method was used to quantify the expression of the aim gene using GAPDH as a normalization control [22].

### Western-blot

Samples were lysed with RIPA lysis buffer containing protease and phosphatase inhibitors (Roche, Germany). The lysates were homogenized and the homogenates were centrifuged at 16,000 g for 20 min at 4°C. The supernatants were collected and protein concentrations were determined. Equivalent amounts of protein were subjected to sodium dodecyl sulfate-polyacrylamide gel electrophoresis and transferred onto a polyvinylidene difluoride membrane (Millipore). The membranes were incubated with specific antibodies against CRT (1∶1500; Abcam), STAT3 (1∶2000; Cell Signaling), phosphorylated STAT3 (Tyr705; 1∶1000; Cell Signaling), MnSOD (1∶1000; Epitomics), β-actin (1∶1000; Santa Cruz Biotechnology), and cytochrome c oxidase subunit IV (COXIV; 1∶2500; Cell Signaling). Blots were visualized with a secondary antibody coupled to horseradish peroxidase (Pierce Biotechnology) and an enhanced chemiluminescence detection system (Pierce Biotechnology). In these experiments, β-actin and COXIV were used as loading controls for the whole cellular and mitochondrial proteins respectively.

### Statistical Analysis

All data are presented as mean ± standard deviation (SD) and were analyzed using SPSS 16.0 software. Analysis of data was performed using one-way analysis of variance test and LSD test. *P*<0.05 was considered statistically significant.

## Results

### Clinical Course

After continuous FZD administration for thirty weeks, most of the rats showed inanimate behavior, decreased physical activity and food intake and an increased rate of breathing. Four out of twenty rats in the model group died, while no rats died in the control and untreated groups. Additionally, fourteen out of twenty rats in the model group were found with pericardial effusion, but no effusion occurred in the control and untreated groups. Peritoneal effusion has not been observed in any groups.

### Body and Heart Weights

**Figure 1:**
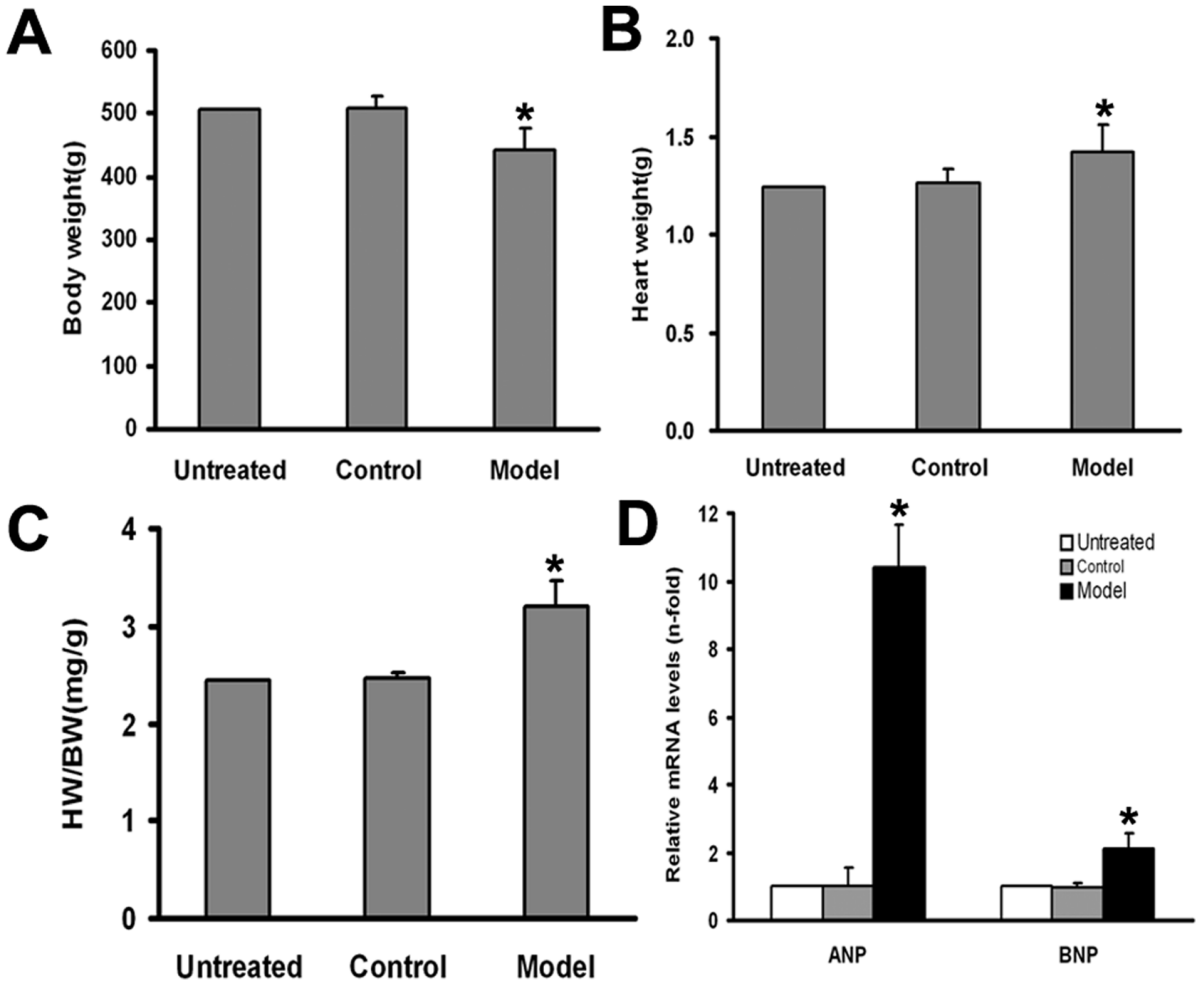
Cardiac hypertrophy in the DCM hearts. Data are mean ± SD (n = 6, 9 and 16 respectively). BW, body weight; HW, heart weight; HW/BW, the ratio of heart weight to body weight; ANP, atrial natriuretic peptide; BNP, brain natriuretic peptide. *P<0.05 versus the control group.

Body and heart weights are shown in Figure 1. The body weight of DCM rats was less than that of the control group (443±34.2 versus 510±18.4 g, *P*<0.05). The heart weight was significantly greater in the model group than the control group (1.42±0.14 versus 1.26±0.07 g, *P*<0.05). The ratio of heart weight to body weight was significantly increased in the model group compared with the control group (3.21±0.27 versus 2.46±0.07 mg/g, *P*<0.05). The body weight, heart weight, and the ratio of heart weight to body weight did not differ between the untreated and control groups (*P*>0.05).

### Echocardiographic, Hemodynamic and Electrocardiographic Parameters

**Figure 2:**
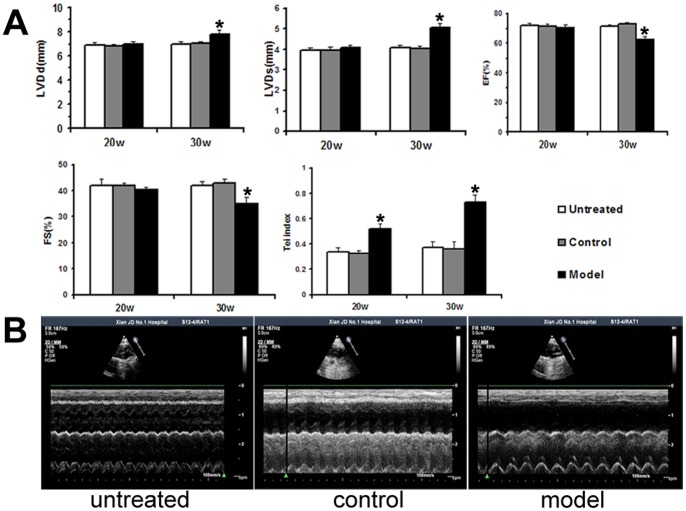
Transthoracic echocardiography analysis. A: Echocardiographic parameters were obtained after twenty and thirty weeks of FZD treatments from the three experimental groups. B: Representative M-mode images of the rats treated with FZD for thirty weeks. Values are mean ± SD (n = 6, 9 and 16 respectively). LVDd, left ventricular end-diastolic dimension; LVDs, left ventricular end-systolic dimension; EF, ejection fraction; FS, fractional shortening; Tei index defined as the sum of the ICT (isovolumetric contraction time) and IRT (isovolumetric relaxation time) divided by the ventricular ejection time. *P<0.05 versus its corresponding control group.

In order to find out for how long the rats should be treated with FZD to establish this model, rat cardiac functions were dynamically monitored through a series of echocardiographic analysis. We did not find any significant differences of cardiac function among the three groups after ten weeks of FZD treatment (data not shown). At twenty weeks of the treatment, LVDd and LVDs in the model group were higher than that in the control group (6.99±0.24 versus 6.78±0.21 mm and 4.08±0.11 versus 3.95±0.16 mm, respectively), but the difference did not reach statistical significance (*P*>0.05, Fig. 2A). After FZD treatment for thirty weeks, echocardiographic analysis revealed that the rats in the model group had enlarged LV systolic and diastolic dimensions and reduced systolic function compared with rats in the control group (Fig. 2). At this time point, hemodynamic measurement obtained through intracardiac catheterization showed significantly reduced LV systolic pressure and impaired dP/dt in the model group compared with the control group (Table 1). Electrocardiographic analysis revealed that heart rate and P-R interval did not differ among the three groups. However, the QRS duration and QαT interval of rats in the model group were significantly longer than that in the control group (Table 2). The rats in the untreated group had similar echocardiographic, hemodynamic and electrocardiographic parameters to those of the control group (*P*>0.05).

**Table 1:** Hemodynamic analysis.

	Untreated(n = 6)	Control(n = 6)	Model(n = 10)
dP/d*t_max_*, mmHg/s	8406±855	8668±723	5258±500*
dP/d*t*_min_, mmHg/s	7463±820	7559±1093	4951±744*
LVSP, mmHg	139±8.86	135±8.49	111±15.8*
LVEDP, mmHg	−1.42±0.33	−1.34±0.45	2.46±0.78*

Data are mean ± SD. dp/dt, first derivative of pressure; LVSP, left ventricular systolic pressure; LVEDP, left ventricular end-diastolic pressure.

* *P*<0.05 versus the control group.

**Table 2:** α-lead electrocardiogram data.

	Untreated(n = 6)	Control(n = 6)	Model(n = 10)
Heart rate	392±22.5	388±27.3	367±71.5
P-R interval (ms)	50.9±1.83	51.8±1.06	51.1±2.44
QRS duration (ms)	16.9±1.45	17.1±1.38	20.2±0.85*
QαT interval (ms)	31.3±1.56	31.5±1.39	35.0±1.42*

Data are mean ± SD. The P-R interval is marked from the beginning of the P-wave to the beginning of the QRS complex. The QRS duration is identified as the time between the upstroke of the R-wave and the point at which the ascending limb of the S-wave crosses the isoelectric line. The QαT interval is identified as the interval between the R-wave upstroke and the apex of the T-wave.

* *P*<0.05 versus the control group.

### Cardiac Morphological Changes

**Figure 3:**
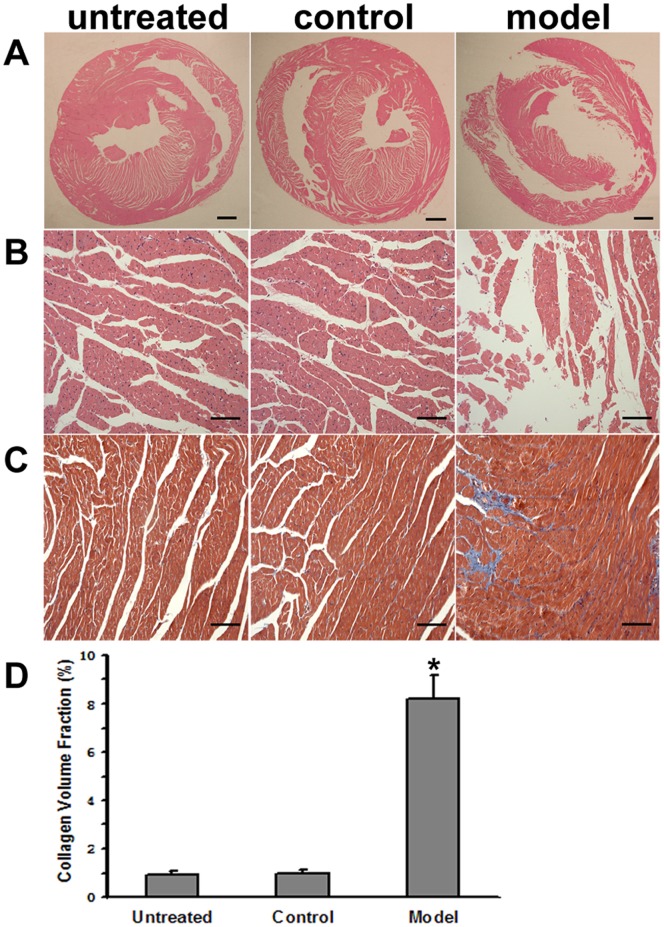
Histopathological changes in the rat hearts. A: Representative macroscopic transversal sections of hearts. The right and left ventricle of DCM hearts are dilated and the LV wall thickness is decreased. Scale bar = 1 mm. B: Photomicrographs show representative myocardial sections stained with HE. Myocardial architecture is intact with cardiomyocytes lined up in order in the untreated and control groups. Myofibrils are widely disintegrated, accompanied by multifocal degeneration and myocytolysis with diffused lymphocytes and monocytes infiltration in the model group. Scale bar = 0.1 mm. C: Photomicrographs show representative myocardial sections stained with Masson’s trichrome. Obvious heart fibrosis is observed in the model group. Scale bar = 0.1 mm. D: Quantitative analysis demonstrated that the collagen volume fraction in the model group (n = 16) was significantly greater than that in the untreated (n = 6) or control group (n = 9). *P<0.05 versus the control group.

Morphological analysis showed that the ventricular dimension was increased and ventricle wall thickness was decreased in FZD induced DCM rats (Fig. 3A). Under optical microscopic observation, focal and diffuse areas of myocardial degeneration characterized by multiple vacuolation of cardiomyocytes, disorganization of myofibrils, disappearance of the myocardial pattern, and homogenization of the sarcoplasm were observed in the model group. However, the cardiomyocytes were regularly arranged with clear myocardial structures in the control and untreated groups (Fig. 3B). Moreover, the DCM hearts showed significant interstitial edematous and fibrosis while little oedema and fibrosis has been observed in the control and untreated groups (Fig. 3C). Further quantitative analysis showed that collagen volume fraction in the model group was significantly greater than that in the control or untreated groups (*P*<0.05, Fig. 3D).

### Mitochondrial Morphology and Function

**Figure 4:**
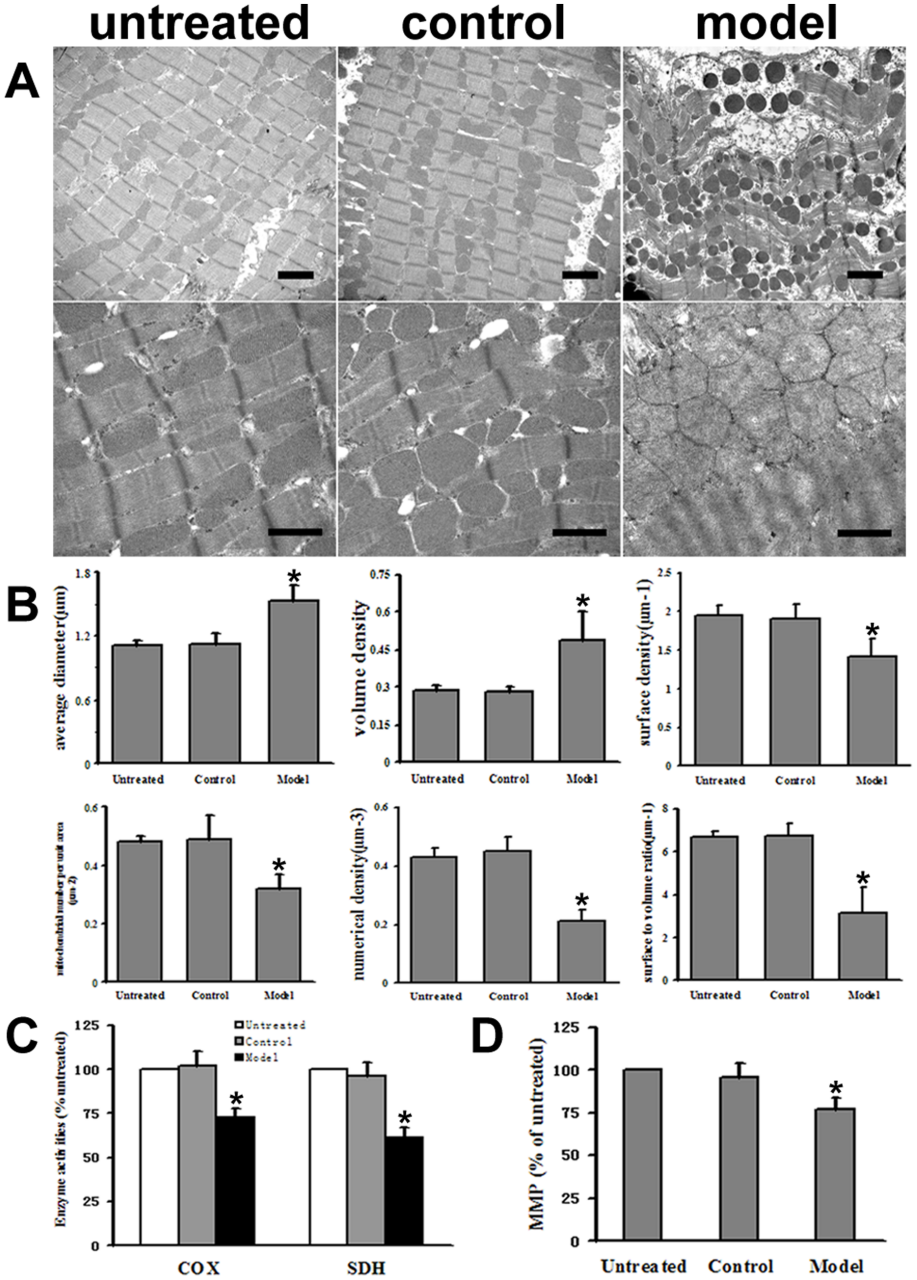
Mitochondria structure and function in the rat hearts. A: Ultrastructure of the rat cardiomyocytes. Normal histological structure of the myocardium was shown in the untreated and control groups. Model group displayed prominent myofilament disarray and rupture, cytoplasmic vacuolization, significant mitochondrial swelling, and focal dissolution of mitochondrial cristae. The upper scale bar = 2 µm, and the lower scale bar = 1 µm. B: Stereological parameters of myocardial mitochondria among the three experimental groups. Data are mean ± SD (n = 6, 6 and 10 respectively). Stereological analysis of cardiac mitochondria from the model group showed notable increases in average diameter and volume density, and remarkable decreases in surface density, mitochondrial number per unit area, numerical density and surface to volume ratio compared with the control values. C: Compared with the control group, COX (Cytochrome c oxidase) and SDH (succinate dehydrogenase) activities of cardiac mitochondria in the model group were significantly decreased. Data are mean ± SD (n = 6, 9 and 16 respectively) D: MMP (mitochondrial membrane potential) level of the freshly isolated cardiac mitochondria in the model group significantly decreased. Data are mean ± SD (n = 6, 9 and 16 respectively). *P<0.05 versus the control group.

Using electron microscopy, we observed morphological changes of cardiomyocyte ultrastructure in the model group, including swelling of mitochondria with fractured or dissolved cristae, vacuolization of the cytoplasm, and dilation of the sarcotubular system with irregular cell nuclei. However, the cardiac ultrastructure in the untreated and control groups showed regular myofibril arrangement, maintained sarcotubular systems, and preserved mitochondria (Fig. 4A).

Stereological analysis of rat cardiac mitochondria from the three groups revealed significant increases in D and Vv in DCM rats compared with the control values (1.53±0.14 versus 1.12±0.11 µm and 0.49±0.11 versus 0.28±0.02, respectively, both *P*<0.05). While the mitochondrial stereological parameters including Sv, N, Nv and Rsv in the model group (1.42±0.24 µm^−1^, 0.32±0.05 µm^−2^, 0.21±0.04 µm^−3^ and 3.17±1.15 µm^−1^, respectively) significantly decreased compared with the control group (1.91±0.19 µm^−1^, 0.49±0.08 µm^−2^, 0.45±0.05 µm^−3^ and 6.75±0.58 µm^−1^, respectively, all *P*<0.05). There were no significant differences of these stereological parameters of mitochondria between the untreated and control groups (Fig. 4B).

Compared with the control group, MMP level of the isolated fresh cardiac mitochondria in the model group significantly decreased (77.1±6.54% versus 95.66±8.57%, *P*<0.05, Fig. 4D). Moreover, the enzyme activities of COX and SDH were lower in the model group compared with the control group (72.7±5.5% versus 102±8.05% and 61.2±5.7% versus 96.3±8.01%, respectively, both *P*<0.05, Fig. 4C). MMP, COX, and SDH did not differ between the untreated and control groups (*P*>0.05).

### Alterations of CRT-STAT3 Signaling Pathway in the Heart of DCM rat

**Figure 5:**
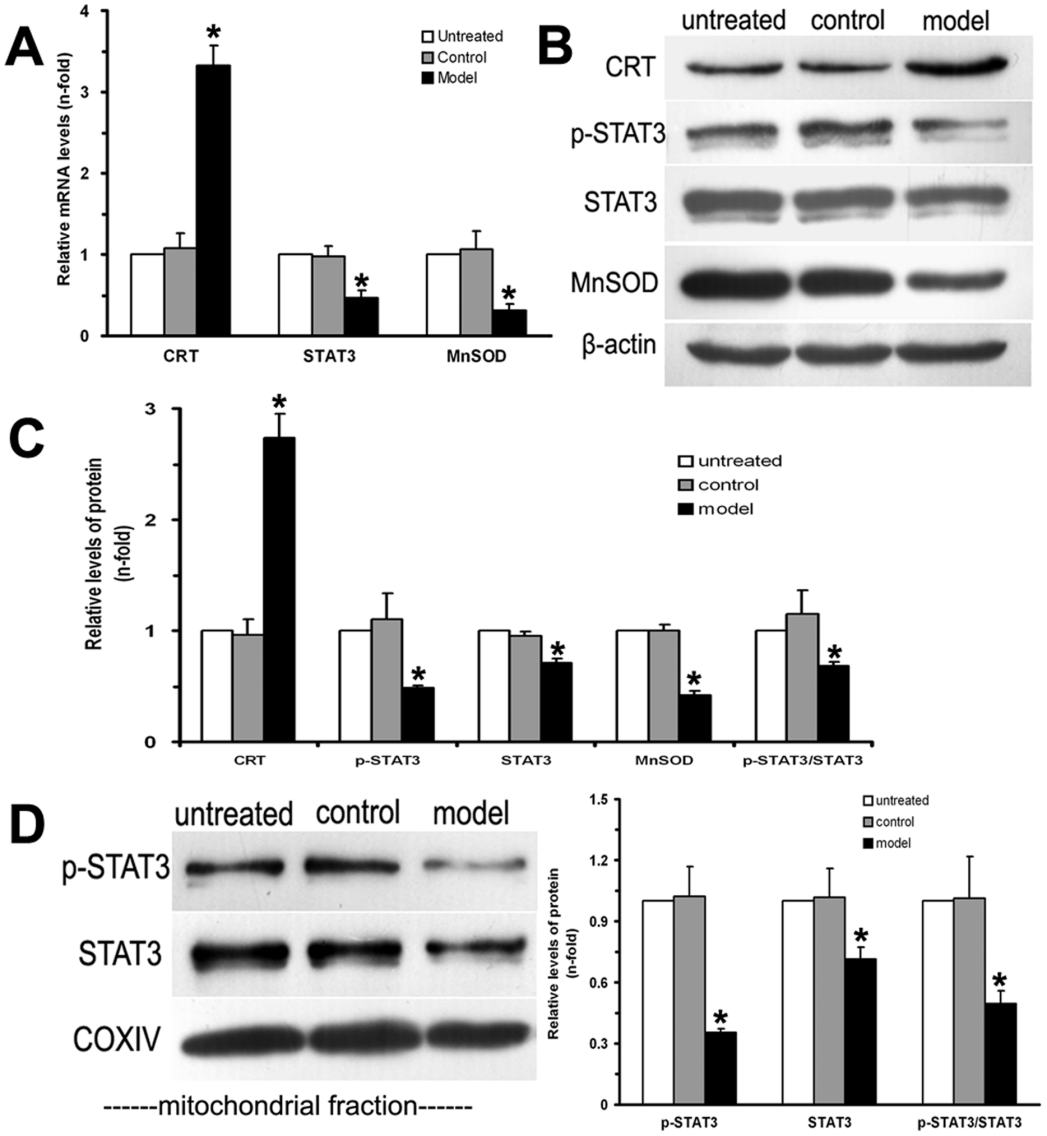
Alterations of CRT-STAT3 signaling pathway in this rat model of DCM. A: Real-time PCR revealed that CRT, STAT3 and MnSOD mRNA expression in the hearts from DCM have changed significantly. Results are expressed as fold increase or decrease over untreated group and GAPDH was served as an internal control (n = 6, 9 and 16 respectively). B: The protein expression of CRT, phosphorylated and total STAT3, and MnSOD in homogenates of rat left ventricular myocardium was determined by western blot. β-actin was used as a loading control. A representative blot is shown. C: Quantification analysis of the protein levels as shown in part B using the quantity one software. D: The protein levels of phosphorylated and total STAT3 in cardiac mitochondria were determined by western blot. COXIV was used as a loading control for the mitochondrial protein. A representative blot is shown in the left panel, and quantitative analysis results are shown in the right panel. The protein expression is represented as the mean ± SD from 6 independent rats per group, and each western blot was performed in triplicate. *P<0.05 versus the control group.

The mRNA expression of CRT, STAT3 and MnSOD were determined by real-time PCR. Compared with the control group, the mRNA level of CRT in the model group was approximately 3-fold higher, while STAT3 and MnSOD mRNA in the model group were approximately 2-fold and 3.4-fold lower respectively (*P*<0.05). There were no differences of CRT, STAT3 and MnSOD mRNA levels between the control and untreated groups (Fig. 5A).

We also analyzed the protein levels of CRT, phosphorylated and total STAT3, and MnSOD using western-blot. The protein level of CRT in the model group was 2.8-fold higher than that in the control group, while phosphorylated STAT3, total STAT3, and MnSOD in the model group were all significantly decreased compared with the control group (*P*<0.05). Moreover, the protein levels of phosphorylated and total STAT3 in cardiac mitochondria of DCM rats were also significantly decreased compared with the control group (*P*<0.05). The ratio of phosphorylated STAT3 to total STAT3 in both the whole cell and mitochondrial fraction of DCM hearts were less than that in the control group (*P*<0.05, Fig. 5B–D). The protein levels of CRT, phosphorylated and total STAT3, and MnSOD did not differ between the control and untreated groups (*P*>0.05).

### Involvement of CRT-STAT3 Signaling Pathway in FZD-induced Mitochondria and Cardiomyocytes Injury in vitro

**Figure 6:**
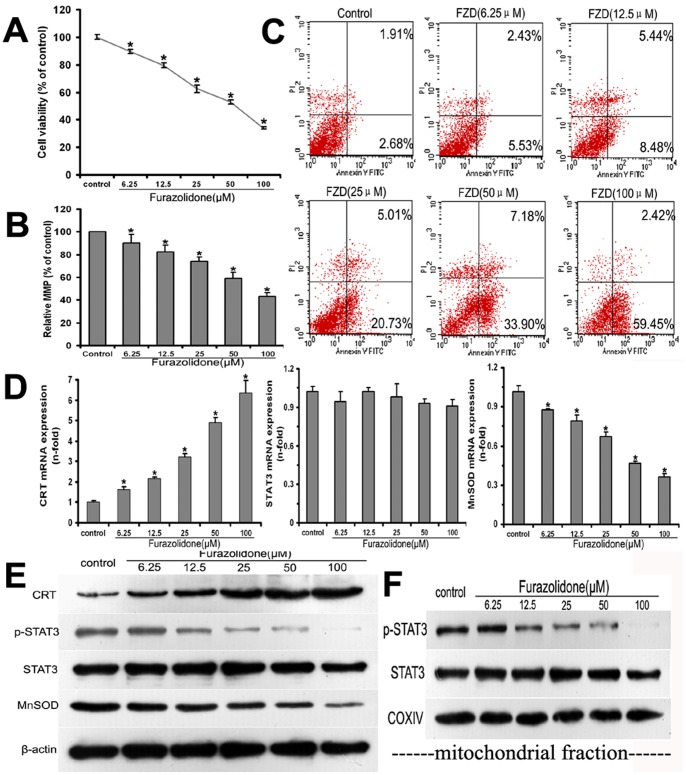
The involvement of CRT-STAT3 pathway in FZD induced mitochondria and cardiomyocytes injury. When neonatal rat cardiomyocytes (NRCMs) were exposed to FZD for 48 h, dose-dependent decreases in cell viability (A) and MMP (B) were observed at the indicated concentration. C: After NRCMs were incubated with FZD for 48 h, annexin V-FITC based apoptosis assay revealed that apoptosis rate was increased in a dose-dependent manner. D: When NRCMs were incubated with FZD for 24 h, real time PCR revealed that FZD regulated the mRNA expression of CRT and MnSOD on a dose-dependent manner, but had no significant effect on the mRNA expression of STAT3. E: After 24 h stimulation with FZD, the protein levels of CRT, phosphorylated and total STAT3, and MnSOD in the whole cell lysates were determined by western blot. β-actin was used as a loading control. A representative blot is shown for each condition. F: Western blot was used to determine the protein expression of phosphorylated and total STAT3 in cardiac mitochondria isolated from NRCMs exposed to FZD for 24 h. COXIV was used as a loading control for the mitochondrial protein. A representative blot is shown for each condition. All statistical data were obtained from 3–5 independent experiments, and represented as the mean ± SD. *P<0.05 versus the control group.

In vitro MTT assay found that cell viability was decreased in a dose-dependent manner when NRCMs were exposed to 6.25–100 µM FZD for 48 h (*P*<0.05, Fig. 6A). Moreover, MMP level was also decreased in a dose-dependent manner when NRCMs were exposed to FZD (*P*<0.05, Fig. 6B). When NRCMs were incubated with FZD at the concentration of 50 µM, cell viability and MMP dropped to approximately 50% of that in the control group. As shown in figure 6C, annexin V-FITC based apoptosis assay revealed that apoptosis rate was increased in a dose-dependent manner when NRCMs were exposed to 6.25–100 µM FZD for 48 h.

After NRCMs were incubated with FZD for 24 h, the expression of CRT and its downstream signaling molecules was detected using real-time PCR and western-blot. The mRNA and protein expression of CRT in NRCMs increased in a dose-dependent manner after cells were incubated with FZD for 24 h, while MnSOD expression decreased (Fig. 6D–E). FZD markedly inhibited STAT3 phosphorylation in both the whole cell and mitochondria as shown in figure 6E and 6F. Notably, FZD had little effect on the mRNA and protein expression of STAT3 in this vitro experiment.

### CRT Silencing Attenuated FZD-induced Mitochondria and Cardiomyocytes Injury in vitro

**Figure 7:**
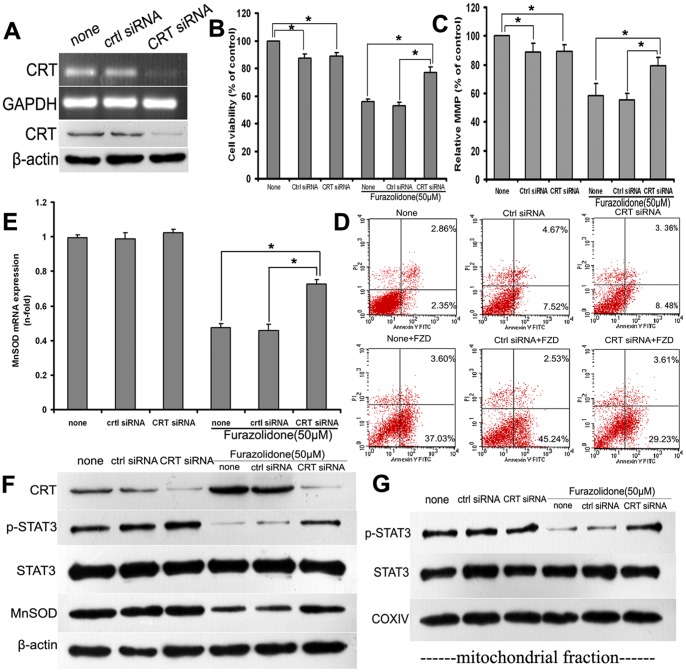
CRT silencing improved FZD-induced injury of mitochondria and cardiomyocytes. A: When cardiomyocytes were transiently transfected for 48 h, the gene silencing effect of CRT was confirmed by RT-PCR (top panel) and western blot (bottom panel). CRT silencing significantly improved the cell viability (B) and MMP (C) of NRCMs stimulated with 50 µM FZD. D: Post-transfection, cardiomyocytes were exposed to 50 µM FZD for an additional 48 h, and annexin V-FITC based apoptosis assay revealed that CRT silencing attenuated cardiomyocyte apoptosis degree. When transfected NRCMs were stimulated with 50 µM FZD for 24 h, real-time PCR revealed that CRT silencing significantly inhibited the decrease of MnSOD mRNA expression (E), and western blot was used to determine the protein levels of CRT, phosphorylated and total STAT3, and MnSOD in the whole cell lysates. β-actin was used as a loading control. A representative blot is shown for each condition (F). Western blot was used to determine the protein expression of phosphorylated and total STAT3 in mitochondria isolated from NRCMs stimulated with 50 µM FZD post-transfection. COXIV was used as a loading control for the mitochondrial protein. A representative blot is shown for each condition (G). All statistical data were obtained from 3–5 independent experiments, and represented as the mean ± SD. *P<0.05.

To elucidate the role of CRT in FZD induced DCM, CRT siRNA was introduced into NRCMs. Cells were harvested at 48 h post-transfection and cell lysate was analyzed by RT-PCR and western-blot. CRT siRNA greatly reduced CRT mRNA and protein expression (Fig. 7A). CRT siRNA was used at a concentration of 100 nM in all subsequent experiments. At forty-eight hours after transient transfection with CRT siRNA, NRCMs were stimulated with 50 µM FZD for an additional 48 h. At this time point, MTT assay revealed that CRT silencing significantly improved the cell viability (*P*<0.05, Fig. 7B). Moreover, MMP level determined by fluorescence microplate reader, and apoptosis rate determined by flow cytometry were both significantly improved compared with the control siRNA transfected cells (*P*<0.05, Fig. 7C–7D).

NRCMs with CRT siRNA transfection were further incubated with 50 µM FZD for an additional 24 h, and the CRT-STAT3 signaling pathway and its downstream signaling molecules were assessed. Only CRT silencing had no significant effect on the protein expression of STAT3 and MnSOD determined by western blot. However, when NRCMs were stimulated with 50 µM FZD post-transfection, CRT silencing significantly attenuated the decrease of phosphorylated STAT3 in both the whole cell and mitochondria (*P*<0.05, Fig. 7F–7G). CRT silencing also significantly inhibited the decrease of MnSOD mRNA and protein expression when NRCMs were stimulated with 50 µM FZD (*P*<0.05, Fig. 7E–7F).

## Discussion

In our study, a rat model of DCM was first successfully induced by a long-term FZD treatment which mimics human DCM. We found that CRT-STAT3 signaling pathway was involved in the progress of DCM in FZD treated rats, associated with serious cardiac mitochondrial damage. The mitochondria and NRCMs injury induced by FZD was dependent on CRT molecule, which inhibits STAT3 phosphorylation.

Several methods have been used to establish animal model for DCM study, including naturally occurring DCM [23], genetically modified animal models [24], [25], autoimmune myocarditis induced DCM [26], chronic rapid pacing induced DCM [27], doxorubicin induced DCM [28], and FZD induced DCM [16]. FZD induced DCM in turkey poult is a common animal model for DCM research [15], [16]. Our previous experiments have shown that long-term treatment of FZD at the dose of 700 ppm also induced serious cardiomyopathy in rats (data not shown). The dose of FZD used in this study is based on our pilot study and the results from turkey poult [15], [16]. In order to reduce the treatment time, we also treated rats with 1500 ppm FZD in our pilot study, and found that treatment with 1500 ppm FZD induced a high mortality in rats due to the systemic side effects of FZD. Our current study confirmed that thirty weeks of FZD treatment at the dose of 700 ppm was appropriate for a rat DCM model.

FZD treated rats had the characteristics of human DCM including significant increases in LVDd and LVDs, decreases in EF and FS, and a hypokinetic and globoid LV [1], as determined by bi-dimensional echocardiography and hemodynamic measurement. Moreover, electrocardiogram study revealed that both QRS duration and QαT interval in DCM rats were significantly longer than the control values, while heart rate and P-R interval did not differ among groups, which are consistent with human DCM [29]. In FZD induced DCM, the phenotype of ventricular dilation, heart weight, and the increased mRNA expression of ANP and BNP indicated that cardiac hypertrophy was also a feature of this DCM model. Moreover, cardiomyocyte hypertrophy and interstitial fibrosis were also present in FZD induced DCM as that in human DCM [30]. Therefore, a rat model of DCM was successfully induced by the long-term FZD treatment, which mimics human DCM.

The accurate mechanism by which FZD induces DCM is still unknown. FZD, as an inhibitor of monoamine oxidase in some species, might cause something akin to catecholamine mediated cardiotoxicity by opposing catecholamine removal [31]. But other evidence suggests that this might not be the cause, as monoamine oxidase inhibition produced by a chemically unrelated compound (tranylcypromine) in turkey poults does not produce cardiomyopathy [32]. Histopathological findings of cardiomyocyte induced by catecholamine were not present in the DCM hearts induced by FZD [31]. Furthermore, our study found that FZD treated rats were not tachycardic during the development of cardiomyopathy.

Is CRT a key signaling molecule in the progress of FZD induced DCM? In this study, the expression of CRT in FZD induced DCM significantly increased, which was consistent with another recent study [7], suggesting the important role of CRT in DCM. The role of STAT3 in heart failure has been carefully studied by many researches [33]. In consistent with previously reported [9], not only was the ratio of phosphorylated STAT3 to total STAT3 significantly decreased, but also the expression of STAT3 and its phosphorylation form were down-regulated in FZD induced DCM hearts. It has been demonstrated that STAT3 is also present in mitochondria [34], and mitochondrial STAT3 possibly contributes to cardioprotection by modulation of mitochondrial electron transport chain and inhibition of permeability transition pore opening [35], [36]. Our research also demonstrated that phosphorylated STAT3, total STAT3, and the ratio of phosphorylation to total STAT3 in the mitochondria of DCM hearts were all significantly decreased. These results suggest that up-regulated expression of CRT inhibits STAT3 phosphorylation. The reason why total STAT3 changed in FZD induced DCM, might be associated with the mutual regulation among the different cells as a whole system which needs further studies.

Increased expression of CRT and the down-regulation of STAT3 phosphorylation might lead to serious cardiac mitochondrial damage in this FZD induced DCM model. Stereological results in cardiac mitochondria of DCM hearts were consistent with the mitochondrial damage evidenced by morphological and functional analysis. All these results demonstrated that cardiac mitochondria in FZD induced DCM were seriously impaired, suggesting the important role of mitochondria in the progress of DCM as previously reported [37], [38]. Moreover, the expression of MnSOD, which is downstream of STAT3, also significantly decreased in the FZD induced DCM hearts as previous studies reported [9], [39]. These data suggests that down-regulated expression and activity of STAT3 induces cardiac mitochondria damage though down-regulating MnSOD expression in DCM heart. Also decreased expression and activity of mitochondrial STAT3 might aggravate mitochondria injury directly.

The CRT-STAT3 signaling pathway has been involved in FZD induced DCM. It is important to discern whether CRT up-regulation is a result of FZD induced DCM, or is a compensatory change to protect against DCM. Further research is needed to study the effects of cardiomyocyte-specific CRT knock down on the cardiac structure and function in FZD-treated animals. Presently, in vitro study in NRCMs was performed to determine the effect of CRT and FZD on NRCMs and mitochondria.

To find out the cytotoxicity concentration of FZD on the NRCMs, a series of preliminary experiments were done using MTT assays. We finally chose 100 µM as the highest concentration to stimulate NRCMs. With the FZD treatment, the cell viability and MMP level in NRCMs decreased, and the apoptosis rate increased, indicating that FZD induces cardiomyocyte and mitochondria impairments. CRT expression was increased in response to FZD in NRCMs, suggesting that CRT is involved in the FZD induced cardiomyocyte and mitochondria impairments. When NRCMs were transfected with CRT siRNA, the cell viability, MMP and apoptosis rate altered a little due to the toxicity of transfection reagent, while the expression of phosphorylated and total STAT3 and MnSOD was unchanged. This result was consistent with one previous study [40]. Exposed to 50 µM of FZD, CRT silencing significantly protected FZD induced cardiomyocyte and mitochondria impairments, demonstrating that CRT plays a protective role on FZD induced DCM. CRT silencing also attenuated the decrease of STAT3 phosphorylation in both the whole cell and mitochondria when NRCMs were incubated with 50 µM FZD, further suggesting that CRT is an upstream signal molecule of STAT3 as an inhibitor and has a key role in the progress of FZD induced DCM.

In summary, the progress of FZD induced DCM might be due to the up-regulated expression of CRT, which inhibits STAT3 phosphorylation in both the whole cell and mitochondrial fraction. Also the increased expression of CRT might down-regulate the expression of MnSOD though inhibiting STAT3 phosphorylation [11], [41]. Not only can hypophosphorylation of mitochondrial STAT3 not protect mitochondria effectively, but also hypophosphorylation of STAT3 in the whole cell attenuates MnSOD expression, finally leading to cardiac mitochondria injury. However, the detailed mechanism of CRT regulation on STAT3 will be further explored in our future studies.
